# Physical Exercise or Activity and Energy Balance or Metabolism in the Context of Health and Diseases

**DOI:** 10.3390/nu15234909

**Published:** 2023-11-24

**Authors:** Laurent A. Messonnier

**Affiliations:** 1 Inter-University Laboratory of Human Movement Sciences, Department of Sport Sciences, University Savoie Mont Blanc, F-73000 Chambéry, France; laurent.messonnier@univ-smb.fr; Tel.: +33(0)-4-79-75-81-85; 2 Institut Universitaire de France (IUF), F-75231 Paris, France

Regular long-lasting physical exercise demands a tremendous amount of metabolic energy [1]. These high exercise-related energy demands may challenge one’s energy balance [2]. While this may be beneficial in cases of overweight and obesity for example [3,4,5], this may be more detrimental in the case of military missions [6] or long-lasting exercises (e.g., ultra-endurance events [7]). Athletes involved in these latter activities, especially women but also men, are particularly exposed to this problem [8,9]. To circumvent/avoid possible deleterious effects, nutritional strategies before, during, or even after exercise can be implemented [10].

In addition, different sources of energy are mobilized during exercise, and the involvement of these different energy sources varies according to exercise duration and intensity [11,12,13,14,15], as well as nutritional/energetic/metabolic status of the subject [16,17]. Metabolic flexibility (i.e., the capacity to switch from one energy source to another) is important for fueling the exercising muscles and, consequently, for athletic performance [18]. Endurance training modifies/improves metabolic flexibility [18], although the latter may be impeded by various factors, including disturbances in energy stores and disease. 

For this Special Issue, scholars were invited to contribute by papers dealing with energy balance or metabolism, metabolic flexibility, and nutritional strategies before, during, or after exercise in the context of health or diseases. 

In an opinion paper, Professor Brooks [19] details how lactate and its shuttling between/within cells and organs is important for supplying energy to active muscles and what it means for sports nutrition, concluding that supplementation with lactate nutrient compounds may constitute a promising nutritional strategy during exercise. 

In his review paper, Doctor Henderson [20] demonstrates how elevated plasma free fatty acid (FFA) concentrations ([FFA]p) constitute a risk factor for metabolic disease (e.g., insulin resistance, non-alcoholic fatty liver disease, type 2 diabetes). This review also addresses how the modulation of [FFA]p via clinical intervention (e.g., chronic caloric restriction, exercise) or clinical troubles/unhealthy behaviors (e.g., sleep deprivation or apnea, cigarette smoking) can be beneficial or detrimental, respectively, for FFA concentrations and their associated/related metabolic diseases.

In a systematic review and meta-analysis paper, Millet et al. [21] address the question of the effects of acute heat or cold exposure on subsequent energy intake (EI) at rest and during exercise in adults. The results of their analysis reveal that, as a whole, EI was slightly increased/decreased after cold/heat exposure, respectively.

In a thorough review of the literature, Brun and colleagues [22] cover fat and carbohydrate oxidation during exercise, along with associated concepts (e.g., cross-over concept, maximal fat/lipid oxidation, lipid oxidation zone). They also examine (i) the physiological relevance of measuring maximal lipid oxidation to provide insights into mitochondrial function and metabolic flexibility and (ii) the determinants of maximal lipid oxidation (e.g., genetics, exercise and training, age, gender, diet, hormones). Emphasis is placed on the physiological effects of exercise training at maximal lipid oxidation in disease (e.g., diabetes, cancer). 

In another review paper, Fournier et al. [23] propose the notion of combining 24 h movement guidelines (which include physical activity, sleep, and sedentary behaviors) with diet/nutritional guidelines to improve health and well-being in adults and children. Indeed, although dietary and movement behaviors appear to be closely related, their interactions deserve to be considered. 

In a mini review, Emhoff and Messonnier [24] explain the physiological relevance of two concepts, namely the lactate metabolic clearance rate (MCR) and the lactate clamp, as well as their applications, to improve the understanding of energy metabolism during exercise. Their mini review also highlights the importance of the lactate MCR in exercise physiology. Indeed, the lactate MCR determines exercise intensity at the maximal lactate steady state/second lactate threshold, that directly influences performance in endurance events. 

In an original research article, Aoki et al. [25] shows that low-intensity exercise in mice induces increases in the plasma levels of glucagon-like peptide-2, resulting in an upregulation of the expression of proteins associated with carbohydrate digestion (disaccharidase sucrase-isomaltase) and absorption (intestinal glucose transporter 2), demonstrating the beneficial effect of low-intensity exercise in the absorption of carbohydrates in the small intestine.

Regarding another original research article contained in this Special Issue, Brož et al. [26] performed a pilot study to characterize the time course of arterialized blood glucose ([glucose]b) in patients with type 1 diabetes during (i) standardized submaximal exercise (50% of individuals’ heart rate reserve), leading to hypoglycemia ([glucose]b ≤ 3.5 mmol/L in 25 to 85 min according to the patient), and (ii) a subsequent 60 min recovery period, which included the oral ingestion of 20 g of glucose at the beginning of the period. 

Messonnier et al. [27] investigated lactate metabolism in Sickle Cell Trait (SCT) carriers in response to high-intensity exercise. Their main findings indicate that (i) the accumulation of muscle and blood lactate was lower, (ii) the muscle content of monocarboxylate transporter 4 (coupled Lactate^−^-H^+^ transporter) was higher, and (iii) the lactate removal ability was greater among the SCT carriers, compared to their control (HbAA) counterparts. 

In a randomized crossover study, Bailey and coworkers [28] studied the short-term (four-day) responses to glycemia (via continuous monitoring using wearable sensors and considering its time course and associated parameters) of 12 overweight and obese adults (men and women, body mass index of 25 to 45 kg/m^2^) in two different conditions: higher and lower amounts of prolonged sitting. 

The screening of patients at risk for skeletal muscle diseases with oxidative impairment constitutes an unresolved challenge. For their paper, Grillet et al. [29] investigated whether intensity-adjusted blood energy substrate levels (lactate, pyruvate) and redox balance markers (lactate/pyruvate and β-hydroxybutyrate/acetoacetate ratios) measured during a cardiopulmonary exercise test could constitute valid biomarkers to select “at-risk” patients. 
